# Self−Assembling Anchorage of Hyaluronic Acid on the Nanoparticle Surface Confers Superiority of Triple Negative Breast Cancer Treatment

**DOI:** 10.3390/pharmaceutics14112461

**Published:** 2022-11-15

**Authors:** Yingpeng Li, Liang Liu, Hongtao Shang, Xuchen Feng, Ni Fan, Jingyu Wang, Yuqi Wu, Yatong Chen, Xinhong Chu, Min Zhong, Yujiao Sun, Hui Fu, Wei Huang, Yunfei Li

**Affiliations:** 1 College of Chinese Materia Medica, Tianjin University of Traditional Chinese Medicine, Tianjin 301617, China; 2 Engineering Research Center of Modern Chinese Medicine Discovery and Preparation Technique, Ministry of Education, Tianjin University of Traditional Chinese Medicine, Tianjin 301617, China; 3 State Key Laboratory of Bioactive Substance and Function of Natural Medicines, Department of Pharmaceutics, Institute of Materia Medica, Chinese Academy of Medical Sciences and Peking Union Medical College, Beijing 100050, China

**Keywords:** *N*-isopropylacrylamide-grafted chitosan, hyaluronic acid, self-assembly, curcumin, CD44 targeting

## Abstract

Triple-negative breast cancer (TNBC) has been listed as one of the most fatal diseases, and no effective targeting treatment is clinically available. Although CD44-targeting hyaluronic acid (HA) has been utilized as targeting ligands in many studies, no facile ways have been developed through HA self-assembly at the nanoparticle surface. Herein, we reported *N*-isopropylacrylamide-grafted chitosan-based nanoparticles self-assembling with HA (HA-NPs) through electrostatic forces and loaded with curcumin (CUR). The HA-NPs displayed pH-responsive properties due to the chemical modification of chitosan, and the preparation process was optimized by central composite design–response surface methodology. HA anchorage confers the vehicle with tumor-targeting capability. HA-NPs displayed more robust effects of inhibiting TNBC primary tumor growth than free CUR and a plain counterpart but without increased systemic cytotoxicity. In addition, in vivo pharmacokinetic studies showed that HA-NPs significantly increased the in vivo residence time of free CUR and improved the bioavailability of CUR. These findings suggested that chitosan-based HA-NPs may provide a feasible and unique strategy to achieve CD44 targeting and enhance its efficacy in vivo for the treatment of advanced TNBC.

## 1. Introduction

Triple negative breast cancer (TNBC) has been featured as a heterogenous disease entity with a high unmet need [1]. To date, TNBC remains the most challenging subtype of breast cancer to treat in the clinic, and no effective targeting therapeutic regimen is available in the clinic. By far, many studies have reported that the overexpression and transformation of the transmembrane glycoprotein cluster of differentiation 44 (CD44) are closely related to various forms of cancer, including TNBC [2,3]. CD44 is a common marker of breast cancer stem cells, and the association between CD44 and decreased disease-free-survival in the clinic has been widely acknowledged [4].

Hyaluronic acid (HA) is a linear and negatively charged glycosaminoglycan polymer. All CD44 isoforms contain HA binding sites in their extracellular region; thus, HA is one of the major ligands for CD44 [5]. Since HA can specifically recognize the CD44 receptor overexpressed on the surface of tumor cells, HA has been extensively studied in cancer targeting therapy [6,7]. The delivery vehicle is often anchored with HA to achieve the targeting capability against overexpressed CD44. Commonly, there are two ways to coat HA at the surface of the delivering vehicle, including electrostatic interaction and covalent conjugation [8]. Chemically maneuvering HA’s structure is usually carried out through the covalent conjugation with three functional groups, namely, a carboxyl group [9], hydroxyl group [10], or acetamido group [11]. The chemical conjugation of HA with the other polymers to obtain the CD44-targeting vehicles has achieved great success [12], but the complicated procedures for synthesis, purification, and molecular characterization or the rising cost restrict its feasibility for researchers without profound chemistry backgrounds. Furthermore, the modified HA may have a weakened ability to recognize CD44. In contrast, the self-assembling method without changing HA’s structure may have some superiorities, such as a facile preparation procedure, high drug loading, no chemical degradation, and agitation [13]. As a positively charged polymer, chitosan (CS) has been extensively utilized to self-assemble with negatively charged HA and form nanoparticles (NPs) for CD44 targeting. However, this HA–CS polyelectrolyte complex coacervate seems more suitable for the encapsulation of hydrophilic biological macromolecules, such as RNA [14,15], protein [16], and nucleic acid [17], than small-molecule drugs. Curcumin (CUR) has been excessively reported to exert its anti-cancer activities via the targeting of cancer stem cells of various origins, including breast cancer [18,19], but the extreme hydrophobicity and relatively small-molecule properties make the HA–CS system weak for CUR delivery.

**Figure 1:**
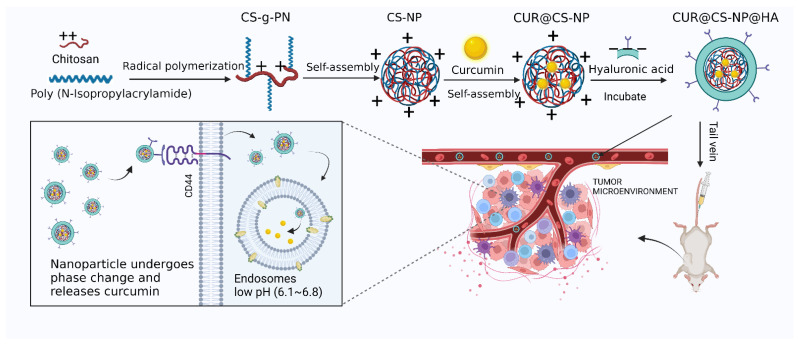
Schematic diagram of NP synthesis and release mechanism.

To this end, a novel CS–HA-based delivery system loaded with CUR was designed and investigated in the present study. Amphiphilic *N*-isopropylacrylamide-grafted CS polymer, after self-assembling a robust NP core, readily interacts with HA to form CD44-targeting NPs (CUR@CS-NP@HA). CUR@CS-NP@HA enriched near tumor cells will specifically recognize CD44 receptors and then mediate tumor cell endocytosis. In the low pH environment of tumor cell endosomes, CS will pH-sensitively release CUR (Figure 1). The in vitro release, cytotoxicity, cell migration, pharmacokinetics, and in vivo antitumor activity of CUR@CS-NP@HA was explored.

## 2. Materials and Methods

### 2.1. Materials

Chitosan (CS), curcumin (CUR), *N*-isopropylacrylamide, and hyaluronic acid (HA) were purchased from Sigma-Aldrich (Shanghai, China). The 4T1 cells were purchased from the National Collection of Authenticated Cell Cultures (China). Fetal bovine serum (FBS), 0.25% trypsin, 0.02% EDTA, penicillin, streptomycin were purchased from Biological Industries (Beijing, China). The 4T1 cells were grown in RPMI medium supplemented with 5% FBS, 1% penicillin/streptomycin, 1 mM sodium pyruvate (Solarbio, Beijing, China), and 0.4 mM glutamine (Solarbio, Beijing, China) and maintained at 37 °C, 5% CO_2_.

### 2.2. Synthesis and Characterization of CS-g-PN

CS-*g*-PN was synthesized successfully by referring to the previously reported studies [20]. The CS with various molecular weights (MWs) (1–50 kDa (low MW CS, CS_LMW_), 50–90 kDa (medium MW CS, CS_MMW_), and more than 90 kDa (high MW CS, CS_HMW_)) were weighed as 250 mg and then dissolved in 100 mL of 0.6% acetic acid solution, respectively. The CS_LMW_ solution was stirred at 80 °C under nitrogen atmosphere for 30 min, and then 1.5 mL of ammonium persulfate solution (2.0 mg/mL) was added, and stirring was continued at 80 °C for another 10 min under nitrogen atmosphere. Finally, 10 mg methylene-bis-acrylamide and 1 g of *N*-isopropylacrylamide were added into the system and reacted for 3 h under the same conditions (Figure S1). The dialysis method was utilized to remove the small molecules and solvents, followed by lyophilization, to obtain the final grafted product CS_LMW_-*g*-PN. CS_MMW_-*g*-PN and CS_HMW_-*g*-PN were prepared by the same procedure, except CS_MMW_ or CS_HMW_ were used as the starting materials. 

FT-IR (Nicolet 6700, Thermo Company, USA) and ^1^H-NMR (Bruker AC (400 MHz) spectrometers were used to characterize the CS_LMW_-*g*-PN, CS_MMW_-*g*-PN, and CS_HMW_-*g*-PN while using CS_LMW_, CS_MMW_, and CS_HMW_ as controls. ^1^H-NMR used 1% deuterated hydrochloric acid as the testing solvent. Differential scanning calorimetry (DSC) was measured by a PerkinElmer Jade DSC (PerkinElmer Analytical Instruments, Shanghai Co., Ltd., Shanghai, China), and the results were analyzed with Pyris software.

### 2.3. Preparation of CUR@CS-NP@HA

The fabrication of CUR@CS-NP@HA was carried out according to a previously reported study [21]. CS_LMW_-*g*-PN, CS_MMW_-*g*-PN, and CS_HMW_-*g*-PN were each dissolved in 1% acetic acid solution, and then the pH value was adjusted to 5.0 with 2 M NaOH. CUR ethanol solution was added into the system and then stirred for 30 min. Finally, 5 min ultra-sonification was utilized to ensure NP formation (CUR@CS-NP). In the HA-coating stage, 15 mL of a 0.5 wt.% dispersion of CUR@CS-NP was mixed with 0.8 mg/mL HA aqueous solution in the same volume, incubated at 4 °C overnight, and ultimately centrifuged under 8000 rpm × 15 min conditions to remove the aggregations, and the obtained upper supernatant was ultracentrifuged (molecular weight cut-off: 100,000) and resuspended by ddH_2_O as curcumin HA CS composite nanoparticle (CUR@CS-NP@HA). The particle size (PS) was determined using a Zetasizer (Nano-ZS, Malvern Instruments Ltd., Worcestershire, UK) at 25 °C. The morphologies of NPs were confirmed by transmission electron microscopy (HT7700, Hitachi, Tokyo, Japan).

### 2.4. Determinations of Drug Encapsulation Efficiency and Drug Loading Capacity

Generally, the NP was ruptured with methanol and ultra-sonicated to make the complete CUR release. The supernatant obtained after centrifugation (12,000 rpm/min × 20 min) was filtered through a 0.22 μm filter membrane and then analyzed by high-performance liquid chromatography (HPLC). The detailed HPLC settings were as follows: the detector was set at 424 nm; the column was a Waters^®^ SPHERISORB^®^ C18 column (250 mm × 4.6 mm, 5 μm), and the column temperature was set at 35 °C; the mobile phase was acetonitrile/4% acetic acid aqueous solution (pH 2.0; *v*/*v*, 45:55); the flow rate was 1.0 mL/min. The encapsulation efficiency (EE)% and drug loading (DL)% were calculated according to Equations (1) and (2):

(1) $$\mathrm{EE}\%=\frac{W_{\mathrm{loaded}}}{W_{\mathrm{initially}\;\mathrm{fed}}}\times 100$$

(2) $$\mathrm{DL}\%=\frac{W_{\mathrm{loaded}}}{W_{\mathrm{loaded}}+W_{\mathrm{excipient}}}\times 100$$

### 2.5. Formulation Optimization of CUR@CS-NP

Since CS_HMW_-NP had the best performance, the associations between the parameter for NP preparation and the physicochemical properties of CUR@ CS_HMW_-NP were further assessed. To optimize NP preparation, a three-factor, three-level Box-Behnken design by Design-Expert 12.0 was performed as illustrated in Table 1. The study design mainly explored three independent variables: CUR/CS-*g*-PN ratio (X_1_, *w*/*w*), pH (X_2_), and CUR dosage (X_3_). The dependent variables were PS (Y_1_), EE (Y_2_), and DL (Y_3_). The sign and value of the quantitative effect represented the tendency and magnitude of the responses, respectively. PS, EE, and DL were input for the establishment of the respective response surface model (RSM).

### 2.6. In Vitro Drug Release Test

The in vitro release profile of CUR@CS-NP was determined by the dialysis method. A PBS solution containing 30% ethanol at different pH values was used as the release medium. Briefly, 3 mL NP solution was put into the dialysis bag (MW cut off 8000–14,000) and then immersed in 50 mL PBS and shaken in a constant temperature water bath at a speed of 100 rpm/min. The samples were collected from the release medium at 0, 0.15, 0.5, 1, 2, 4, 6, 8, 10, 12, and 24 h and the filtered through a 0.22 μm filter membrane, in which the concentration of CUR was detected by HPLC. Furthermore, the same volume of fresh buffer medium was supplemented into the dialysis medium.

### 2.7. In Vitro Cytotoxicity Assay

The MTT assay was performed to evaluate the in vitro cytotoxicity. Briefly, cells were seeded on 96-well plates (3–5 × 10^3^ cells/well in 100 μL of complete culture medium). Different concentrations of polymers or NPs loaded with CUR were added into the wells 24 h after cell seeding and incubated with cells for 48 h; saline or dimethyl sulfoxide (DMSO) was used as a positive control. At the end of incubation, 50 μL of the MTT reagent (5 mg/mL) was added to each well and incubated for 4 h. Then, 200 μL of DMSO was added to each well and incubated for another 1 h. The plate was recorded using a microplate reader (SpectraMax i3x, Molecular Devices, Sunnyvale, CA, USA) at the wavelength of 570 nm. The inhibition of relative cell growth was determined by the following formula: Cell Viability (%) = (Absorbance at 570 nm of NP treated cells)/(Absorbance at 570 nm of control-treated cells) × 100.

### 2.8. Cellular Uptake Study

Briefly, 4T1 cells were seeded into 6-well plates at a density of 1.0 × 10^4^ cells/well. The cells were treated by the free CUR group, the CUR@CS_LMW_-NP group, the CUR@CS_MMW_-NP group, the CUR@CS_HMW_-NP group, and the CUR@CS-NP@HA group for 6 h. After that, the upper culture medium was aspirated off, and the cells were washed with cold PBS solution. The cells were fixed with 200 μL of 4% cell fixative for 10 min and then incubated with DAPI solution for 10 min. The fluorescence intensity of CUR was observed with an inverted fluorescence microscope, and the quantitative analysis was performed with Image J software.

### 2.9. Cell Migration Assay

Briefly, 4T1 cells at the logarithmic growth stage were digested with 0.25% trypsin. The single cells were collected, resuspended in RPMI-1640 medium, and seeded in 6-well plates (5 × 10^5^ cells/well) at 37 °C. After 24 h incubation, a line was evenly drawn at the plate bottom, and then the treatment started. The experimental groups were set as blank control group, free CUR group, CUR@CS_HMW_-NP group, and CUR@CS-NP@HA group. The applied CUR concentration was 5 μM. Representative photos were taken under an inverted microscope at 0, 4, 12, and 24 h, and the scratch areas were calculated using Image J software.

### 2.10. In Vivo Study

#### 2.10.1. Pharmacokinetic Study

The pharmacokinetic study protocol was reviewed and approved by the Animal Ethics Committee at Tianjin University of Traditional Chinese Medicine and followed the animal care guidelines of the Laboratory Animal Management Committee. SD rats weighing 180–220 g were obtained from Beijing HFK Experimental Animal Culture Co., Ltd. (Beijing, China). Briefly, 18 SD rats were randomly divided into three groups with 6 rats in each group: CUR solution, CUR@CS-NP, and CUR@CS-NP@HA. Upon systemic administration at a dosage of 1.5 mg CUR/kg, blood collections from the rat orbital veins were performed at 0.033, 0.083, 0.167, 0.25, 0.5, 1, 2, 4, 6, 8, 10, 12, and 24 h, and 0.5 mL blood samples were centrifuged at 5000 rpm, 4 °C, for 10 min in heparinized tubes. Subsequently, the upper layer plasma samples were taken (100 μL) and placed in a 1.5 mL centrifuge tube containing 300 μL methanol, followed by 30 s vortexing and 5000 rpm × 20 min centrifugation at 4 °C. The supernatant was collected, and the fluorescence intensity was determined with a multi-functional microplate reader (excitation: 436 nm; emission: 530 nm).

#### 2.10.2. Evaluation of Antitumor Activity

The antitumor study protocol was reviewed and approved by the Animal Ethics Committee at Tianjin University of Traditional Chinese Medicine and followed the animal care guidelines of the Laboratory Animal Management Committee. Six-week-old Balb/c mice were obtained from Beijing HFK Experimental Animal Culture Co., Ltd. (Beijing, China). Mouse mammary tumors were established by directly injecting 0.5 million 4T1 cells into the third fat pad at the left flank. The treatment started when mammary tumor volumes reached about 100 mm^3^. Free CUR, CUR@CS-NP, and CUR@CS-NP@HA were intravenously administered every two days at the dosages of 8 mg CUR/kg body weight; saline was set as the negative controls. Tumor volumes were measured every day. After euthanasia, the liver, heart, spleen, lung, and kidney were collected and examined by HE stains [22].

#### 2.10.3. Quantitative Polymerase Chain Reaction (qPCR)

Tissue total RNAs were extracted using Trizol reagent (Invitrogen, Waltham, MA, USA), and RNA concentrations were measured using NanoDrop^TM^ One (Thermo Scientific, Waltham, MA, USA). The cDNA was synthesized using the SuperScript^TM^ II RT (Invitrogen) for qPCR amplification, which was carried out in an ABI QuantStudio 3 System using the ABI TaqMan gene expression assay. The GAPDH level was analyzed and used to normalize specific gene mRNA expression levels, as described in our previous study [23]. The primer sequences (5′→3′) in the PCR are shown in Table 2.

#### 2.10.4. Blood Routine Analysis

Before the mice euthanasia, 1 mL of venous blood was drawn, and the average hemoglobin concentration (MCHC), hemoglobin (HGB), average erythrocyte volume (MCV), red blood cell specific volume (HCT), red blood cell distribution width (RDW), mean corpuscular hemoglobin (MCH), and red blood cell (RBC) and white blood cell (WBC) contents in the blood were measured and analyzed by the blood routine analyzer.

### 2.11. Statistical Analysis

The optimization design of CUR@CS-NP and the data were analyzed by Design-Expert software (12.0 version, Stat Ease, Inc., Minneapolis, MN, USA). The results were presented as means ± standard deviations (SDs). Statistical analysis of results was performed by one-way ANOVA using SPSS software (17.0 version, SPSS Inc., Chicago, IL, USA). The statistical significance was set at *p* < 0.05.

## 3. Results and Discussion

### 3.1. Characterization of CS-g-PN

CS is a natural alkaline polysaccharide, and its protonated amine group can interact with the negatively charged plasma membrane to increase cell membrane permeability, which facilitates drug entry into tumor cells. Thus, CS is an ideal biodegradable material for drug delivery.

Due to a wide molecular weight (MW) distribution, CS can be categorized generally as low MW CS (MW < 50 kDa), medium MW CS (50 kDa < MW < 90 kDa), and high MW CS (MW > 90 kDa). It has been found that the CS MW affects its physical and chemical properties. CS_LMW_ has low viscosity and good granulation performance, while CS_HMW_ is viscous and easily aggregated but has good entrapping ability for drugs. Three kinds of CS-grafted *N*-isopropylacrylamide polymers (low, medium, and high MW) were synthesized by radical polymerization. FT-IR and ^1^H-NMR were used to characterize CS-*g*-PN. Furthermore, the dynamic light scattering method was used to determine the sizes of CS_LMW_-NP, CS_MMW_-NP, and CS_HMW_-NP, which could suggest the MW’s impact on the particle size.

**Figure 2:**
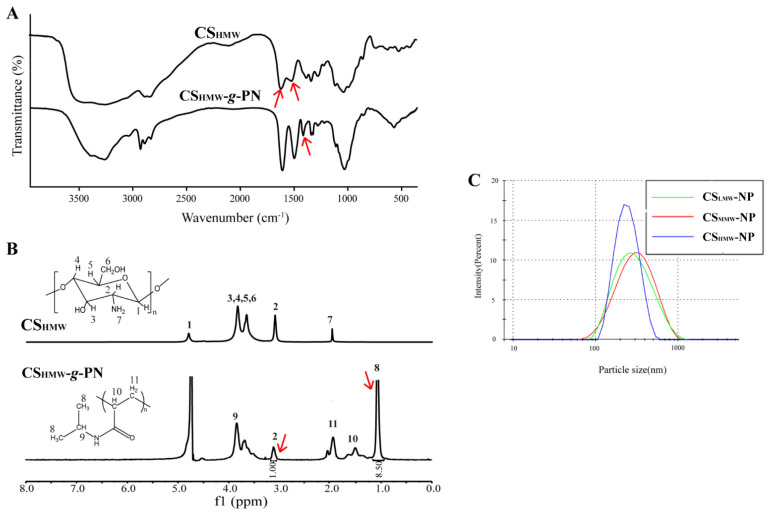
Characterization of CS-*g*-PN. (**A**) FT-IR spectra of CS_HMW_ and CS_HMW_-*g*-PN. The three red arrows illustrate a newly arisen peak in CS_HMW_-*g*-PN and two disappeared peaks in CS. The upper two arrows represent peaks at 1640 and 1520 cm^−1^, the lower single arrow represents the peak at 1385 cm^−1^. (**B**) ^1^H-NMR spectra of CS_HMW_ and CS_HMW_-*g*-PN. Two arrows represent peaks at 3.1 and 1.1 ppm. (**C**) Particle size distribution of CS_LMW_-NP, CS_MMW_-NP, and CS_HMW_-NP.

The successful synthesis of CS-*g*-PN polymers was characterized by FT-IR and ^1^H-NMR methods, referring to a previous study [20]. CS_LMW_, CS_MMW_, and CS_HMW_ all displayed similar FT-IR spectra patterns (Figure 2A and Figure S2A), while CS_LMW_-*g*-PN, CS_MMW_-*g*-PN, and CS_HMW_-*g*-PN showed different FT-IR spectra patterns compared to their counterparts. The spectrum of CS-*g*-PN displayed new peaks at 1385 cm^−1^ compared with that of CS (Figure 2A), suggesting the presence of isopropyl groups from *N*-isopropylacrylamide in CS-*g*-PN. In addition, the absorption bands at 1640 and 1520 cm^−1^ in the figure were attributed to the C=O stretching vibration on the amide bond. In the FT-IR spectrum of CS-*g*-PN, the peak intensity of the absorption band was enhanced. This phenomenon confirmed the presence of more amide bonds on CS, and the successful polymerization of CS with *N*-isopropylacrylamide.

^1^H-NMR also confirmed that *N*-isopropylacrylamide was successfully grafted onto CS. In line with the FT-IR spectra, the ^1^H-NMR of CS_LMW_-*g*-PN, CS_MMW_-*g*-PN, and CS_HMW_-*g*-PN also showed a similar pattern (Figure 2B and Figure S2B). The ^1^H-NMR spectrum (Figure 2B) of CS showed typical g proton peaks on the carbon of CS (3.5–4.0 ppm). However, unlike the CS spectrum, the ^1^H-NMR spectrum of CS-*g*-PN displayed new peaks from methyl groups at 1.1 ppm. Furthermore, the two peaks at 1.2–2.0 ppm (>CH-CH_2_−) also identified the presence of *N*-isopropylacrylamide. The grafting ratios of CS-*g*-PN were evaluated by the ratio of the integral area at δ 1.1 ppm (the methyl group of *N*-isopropylacrylamide) to that at δ 3.1 ppm (>CH-NH_2_ of CS). The ratio of grafting degree of *N*-isopropylacrylamide on CS_LMW_-*g*-PN, CS_MMW_-*g*-PN, and CS_HMW_-*g*-PN was determined to be 8:20:3.

When presenting at room temperature and in a neutral pH condition, the hydrophobic domains of isopropyl groups in CS-*g*-PN exhibit a reversible phase transition and force the loss of hydrogen bonds and the release of water [24,25,26,27,28], which ultimately confers CS-*g*-PN amphiphilic properties to self-assemble into a “core–shell” nanoparticle. We prepared blank NPs by self-assembly by dissolving CS-*g*-PN (low, medium, and high MW CS) in water. As shown in Figure 2C, the size and PDI of CS_HMW_-NP without any payloads were relatively smaller. This may be attributed to the more hydrophobic core of CS_HMW_-NP, which was tightly arranged and enhanced the self-assembly ability of NPs.

### 3.2. Optimization and Validation

In the pilot study, the pH, the CUR/CS-*g*-PN ratio (*w*/*w*), and the dosage of CUR were found to have significant effects on EE, DL, and PS. Based on the range of the single factor test, the optimal CUR@CS-NPs formulations were determined by PS, EE, and DL combined with the results of the effect surface.

After software processing and analysis, the corresponding relationship was determined with a quadratic polynomial, as shown in Equation (3):

Y_1_ = 238.38 − 68.19X_1_ − 23.31X_2_ + 33.2X_3_ − 2.47X_1_X_2_ − 11.07X_1_X_3_ − 11.09X_2_X_3_ + 69.51X_1_^2^ − 1.62X_2_^2^ + 4.65X_3_^2^ Y_2_ = 71.53 + 2.19X_1_ + 0.55X_2_ + 0.32X_3_ − 0.95X_1_X_2_ + 2.65X_1_X_3_ + 2.65X_2_X_3_ − 2.46X_1_^2^ − 6.11X_2_^2^ − 8.39X_3_^2^ Y_3_ = 9.52 − 2.01X_1_ + 0.09X_2_ + 0.03X_3_ − 0.22X_1_X_2_ + 0.04X_1_X_3_ + 0.32X_2_X_3_ + 0.12X_1_^2^ − 0.88X_2_^2^ − 1.11X_3_^2^ (3)

where Y_1_–Y_3_ is the dependent variable (the PS, EE, and DL of CUR@CS−NPs), X_1_–X_3_ are the independent variables (the pH value, CUR/CS-*g*-PN ratio (*w*/*w*), and dosage of CUR, respectively).

**Figure 3:**
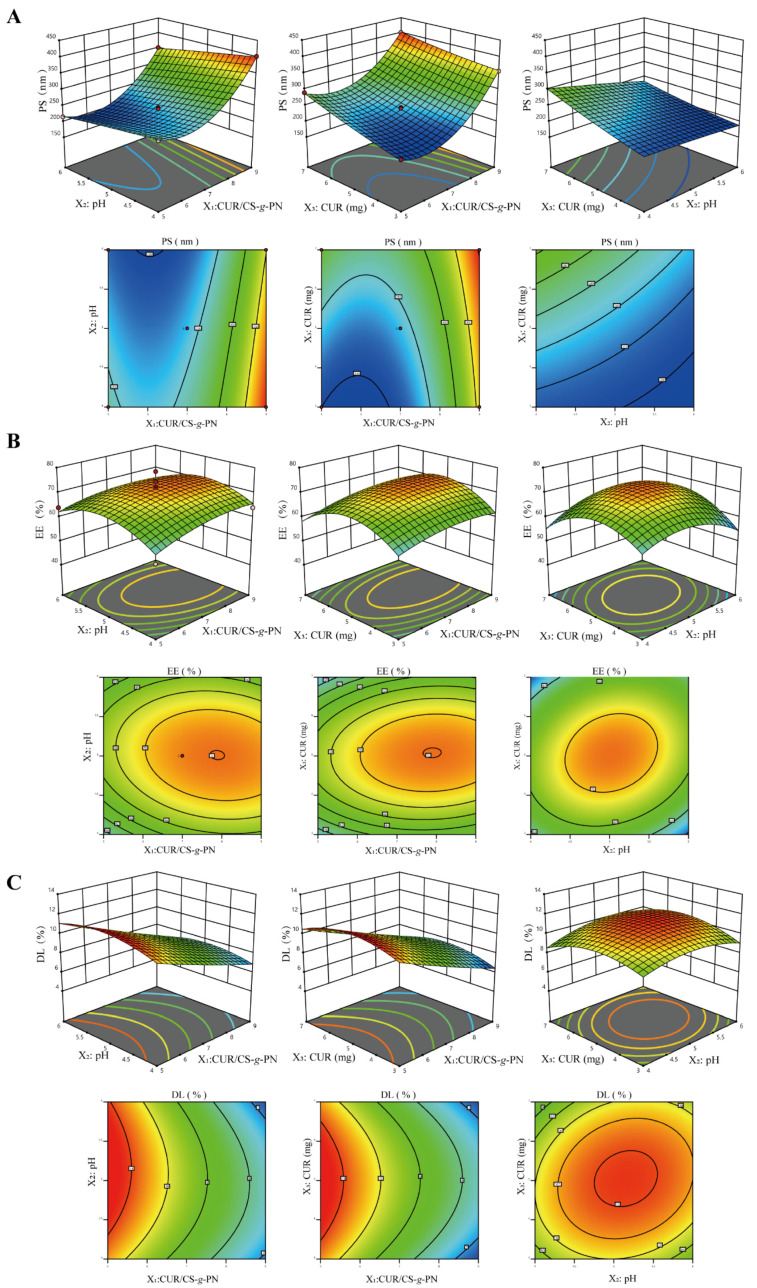
The three-dimensional response surface plots and contour maps for PS, EE, and DL. (**A**) Central composite design-response surface for the effect of pH, CUR/CS-*g*-PN ratio, and CUR dosage on PS. (**B**) Central composite design-response surface for the effect of pH, CUR/CS-*g*-PN ratio, and CUR dosage on EE. (**C**) Central composite design-response surface for the effect of pH, CUR/CS-*g*-PN ratio, and CUR dosage on DL.

Therefore, the best-fit model for the responses was the quadratic model. ANOVA was utilized to evaluate the significance of the quadratic model terms on response. The pH value, the CUR/CS-*g*-PN ratio (*w*/*w*), and the dosage of CUR had significant effects on the response (*p* < 0.05). Response surfaces were optimized for multivariate analysis and plotted on a 3D model graph (Figure 3). Through software analysis, we obtained an optimal formulation with a pH of 5.2, a CUR dosage of 5.0 mg, and a CUR/CS-*g*-PN ratio (*w*/*w*) of 1:5. The predicted values of PS, EE, and DL of the CUR@CS-NP prepared by the optimized formulation were 229 nm, 67.86%, and 11.65%, respectively. These data suggest that under an optimal formulation, most of CUR can be encapsulated in NPs. To verify the validity of the designed model, we prepared CUR@CS-NP according to the optimal formulation and measured the actual PS, EE, and DL. As shown in Table 3, the measured values of each index were in line with the predicted values, indicating that the method is effective and reliable and can be used for further experiments.

### 3.3. Characteristics of CUR@CS-NP

**Figure 4:**
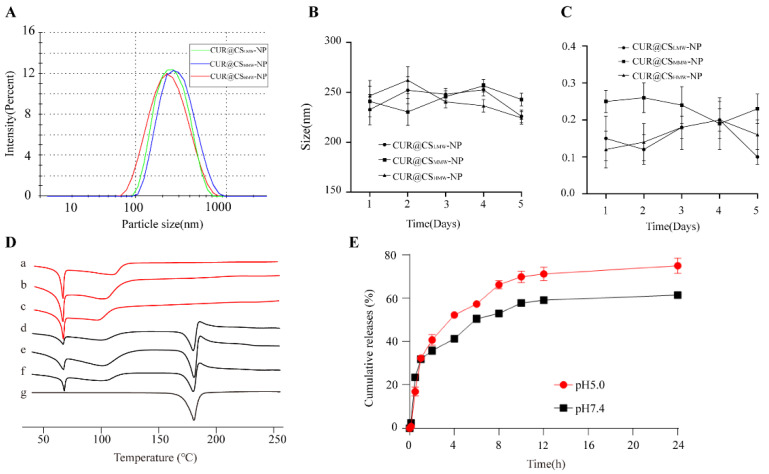
Characterization and in vitro drug release evaluation of CUR@CS-NP. (**A**) PS distribution of CUR@CS_LMW_-NP, CUR@CS_MMW_-NP, and CUR@CS_HMW_-NP. (**B**) PS stability of CUR@CS_LMW_-NP, CUR@CS_MMW_-NP, and CUR@CS_HMW_-NP. (**C**) PDI stability of CUR@CS_LMW_-NP, CUR@CS_MMW_-NP, and CUR@CS_HMW_-NP. (**D**) Differential scanning calorimetry: (a–c) CUR@CS_LMW_-NP, CUR@CS_MMW_-NP, CUR@CS_HMW_-NP; (d–f) physical mixture of blank CS_LMW_-NP, CS_MMW_-NP, and CS_HMW_-NP with CUR; (g) CUR. (**E**) In vitro release profile of CUR@CS_HMW_-NP.

Due to the self-assembly properties, the polymers automatically form a hydrophobic cavity, which is particularly suitable as a container for hydrophobic drugs such as CUR. CUR@CS_LMW_-NP, CUR@CS_MMW_-NP, and CUR@CS_HMW_-NP were prepared by the optimized formulation in Section 3.2. The PS, EE, and DL are shown in Table 4. It was found that CUR@CS_HMW_-NP had the smallest particle size (Figure 4A) and the largest encapsulation efficiency and drug loading capacity, so CUR@CS_HMW_-NP was selected for the subsequent experiments for pharmaceutical investigation. The reason is that the self−assembly performance of CS_HMW_-*g*-PN was more stable, and the ability to encapsulate CUR was stronger. Moreover, the particle size and PDI of CUR@CS_LMW_-PN, CUR@CS_MMW_-NP, and CUR@CS_HMW_-NP did not change significantly within 5 days (Figure 4B,C), indicating the good stability of our prepared NPs.

To confirm the successful entrapment of CUR in NPs, we performed DSC detection on CUR, and a physical mixture of blank CS-*g*-PN and CUR, and CUR@CS-NP. In Figure 4D, the CUR melting endothermic peak appeared at 185 °C, which is indicative of CUR presence as a crystalline form. In the physical mixture of blank CS-*g*-PN and CUR, two melting endotherms could be observed at 65 °C and 185 °C. The presence of an endothermic peak (185 °C) indicated that CUR existed as a crystalline form and was not successfully entrapped into CS-*g*-PN. Of note, CUR@CS-NPs have no endothermic peak at 185 °C, which indicated that CUR has been successfully embedded in the NPs as an amorphous form.

Interestingly, CUR@CS-NP showed stimuli-sensitive properties to a certain extent in the in vitro release experiments. The in vitro release profile studies of CUR@CS_LMW_-NP, CUR@CS_MMW_-NP (Figure S3), and CUR@CS_HMW_-NP (Figure 4E) were performed in release media at pH 7.4 and 5.0. In the pH 5.0 release medium, the cumulative release rate of the NPs within 24 h (around 75%) was higher than that in the pH 7.4 release medium (around 55%). This pH-sensitive property may be attributed to the multiple amine groups in the CS structure. CS is prone to protonation in an acidic environment, and the protonated amine groups repel each other, causing the NPs to collapse and release the drug. Given that tumor cells have a relatively lower pH due to anaerobic metabolism, the pH-sensitive property of CS is good for the drug release at desired sites.

### 3.4. Antitumor Activity In Vitro

While CS-based drug carriers can release drugs around tumor tissue in a higher proportion, HA enables NPs to arrive at the tumor tissue by targeting the CD44 receptor on the surface of tumor cells.

**Figure 5:**
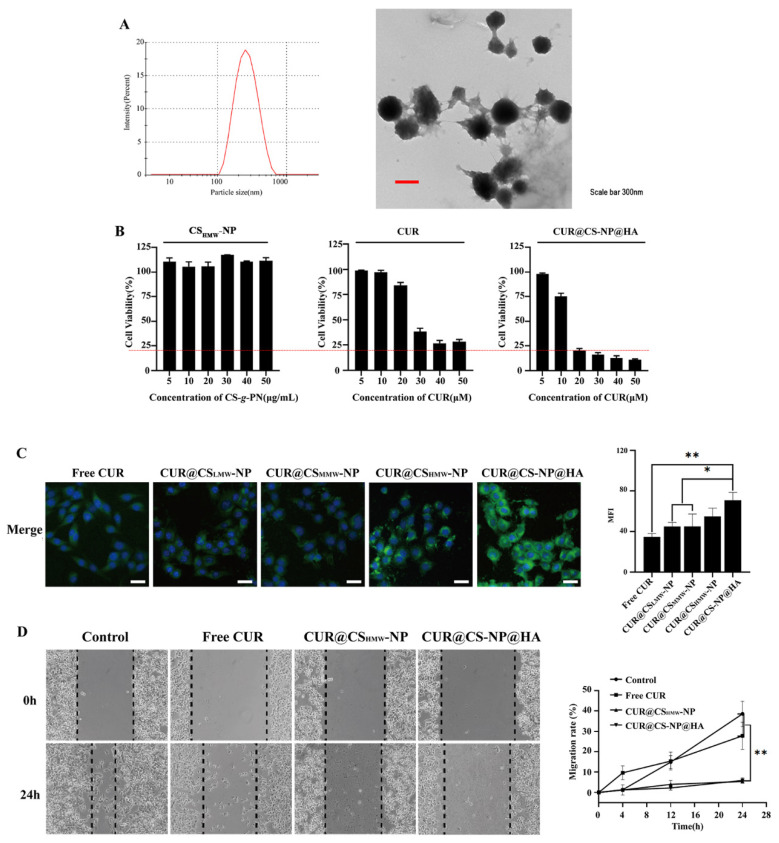
In vitro cell assay for the HA-anchoring NPs fabricated based on CS_HMW_-*g*-PN polymer. (**A**) Particle size distribution and TEM image of CUR@CS-NP@HA. (**B**) Cytotoxicity of vehicle control, CUR, and CUR@CS-NP@HA. (**C**) Uptake of CUR by 4T1 cells (scale bar = 20 μm) (* *p* < 0.05). (**D**) The migration of 4T1 cells treated with NPs (* *p* < 0.05; ** *p* < 0.01).

The surface of HA is negatively charged, while the surface of CS is positively charged, so HA can easily attach at the surface of the CS core through electrostatic forces. The above-discussed experiments proved that CUR@CS_HMW_-NP has suitable particle size and high drug loading, so we chose CUR@CS_HMW_-NP as the NP core and finally obtained CUR@CS-NP@HA. As shown in Figure 5A, the particle size of CUR@CS-NP@HA was around 250 nm, and TEM was used to identify its spherical morphology. As shown in Figure S4, the sizes of CUR@CS-NP@HA in ddH_2_O showed a consistent trend within 5 days, implicating the good stability. Additionally, the zeta-potential of CUR@CS-NP@HA is included in Figure S5. Due to the HA anchorage, the surface charge decreased from 6.9 mV to −1.6 mV. Nevertheless, the repulsive forces originating from the negative surface charge (−1.6 mV) conferred CUR@CS-NP@HA colloidal stability and prevented the aggregation of particles. Like CUR@CS-NP, CUR@CS-NP@HA also showed stimuli-sensitive properties implicated in the in vitro release study (Figure S6), in which lower pH stimuli could accelerate the CUR release. FT-IR spectroscopy of CS-NP@HA (Figure S7) showed that compared with CS-*g*-PN, the amide bond absorption peak of CS at CS-NP@HA shifted to 1642 cm^−1^, and the C=O (−COOH) symmetric stretching vibration peak of HA shifted from 1413 cm^−1^ to 1414 cm^−1^, both of which indicated that HA interacts with CS-*g*-PN.

An MTT assay was utilized to evaluate the capability of CUR@CS-NP@HA to inhibit the proliferation of tumor cells. In all applied concentrations, the blank NPs had no obvious toxicity to 4T1 cells, which is indicative of good biocompatibility (Figure 5B and Figure S8A). The antiproliferation effects of CUR@CS_LMW_-NP, CUR@CS_MMW_-NP, and CUR@CS_HMW_-NP on 4T1 cells were concentration-dependent, and the viability of 4T1 cells gradually decreased with the increase in CUR concentration (Figure S8B). Compared to the blank vehicle and non-targeting NPs, CUR@CS-NP@HA significantly inhibited the viability of 4T1 cells and could widen the therapeutic window of CUR on 4T1 cells (Figure 5B). Additionally, compared to the IC_50_ value of free CUR of 23.61 μM, the IC_50_ value of CUR@CS-NP@HA was determined to be 11.6 μM. As such, CUR@CS-NP@HA can widen the therapeutic window of CUR, which is particularly useful in real clinics.

We sought to explore the underlying mechanism of CUR@CS-NP@HA in augmenting the antiproliferation efficacy of CUR. The enhanced cellular uptake may be one of the important contributors. A six-hour cellular uptake experiment showed that the fluorescence intensity of CUR from the CUR@CS-NP@HA group was two-fold higher than that of the free CUR group (Figure 5C), while the other three plain NPs had modest effects on the increase of CUR cellular uptake. The carboxyl group in the HA molecular backbone has the ability to recognize the CD44 receptor of breast cancer cells and mediate the endocytosis of NPs by tumor cells. After NPs enter tumor cells, CS undergoes a pH response in the low pH environment of endosomes in tumor cells and swells to release CUR.

CUR is able to diminish the proliferation, metastasis, and invasion of tumor cells and promotes the apoptosis of tumor cells by blocking various cell signaling pathways. The effects of CUR@CS_HMW_-NP and CUR@CS-NP@HA on cell migration ability were investigated. Both CUR@CS_HMW_-NP and CUR@CS-NP@HA groups had the lowest mobility after 24 h (Figure 5D and Figure S9), which disclosed the potency of CUR@CS-NP@HA to inhibit the metastasis of 4T1 breast cancer cells.

### 3.5. Pharmacokinetic Studies

Free CUR molecules represent poor water solubility and low oral bioavailability, greatly restricting their clinical applications [29]. The pharmacokinetics study was performed to explore whether CS-NP@HA encapsulation could solve these CUR problems. 

**Figure 6:**
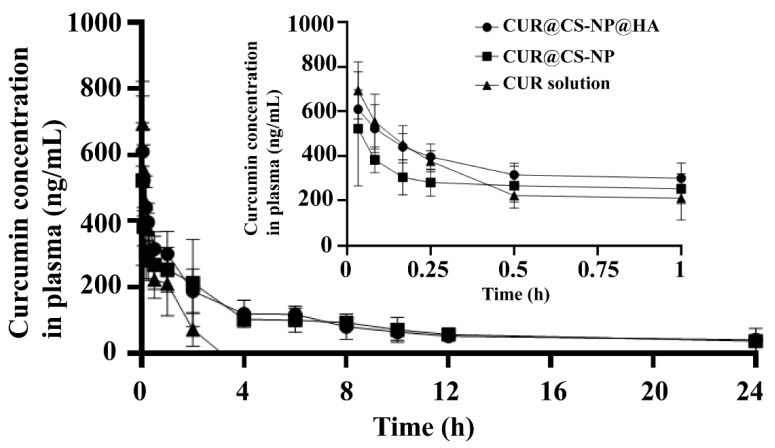
Time–mean drug plasma concentration profiles of CUR@CS-NP and CUR@CS-NP@HA fabricated by CS_HMW_-*g*-PN polymer in rats (1.5 mg/kg). Free CUR was used as the control.

In the present study, the plasma concentrations of CUR in rat plasma were determined by the fluorescence method, and the mean plasma concentration–time curves of CUR in rat plasma (Figure 6) over a 24 h period following tail vein injection of 1.5 mg/kg of free CUR, CUR@CS-NP, and CUR@CS-NP@HA solutions were plotted. Free CUR had a short half-life, which was undetectable 4 h after administration; on the contrary, CUR still presented from 4 h to 24 h post administration in both CUR@CS-NP and CUR@CS-NP@HA groups. Moreover, as shown in Table 5, compared with free CUR, the AUC of CUR@CS-NP and CUR@CS-NP@HA increased by 6.24 and 3.94 times, respectively, the MRT increased by 76.30 and 15.80 times, respectively, and t_1/2_ was extended by 21.59 and 15.80 times, respectively, as their CLs decreased.

As CUR is unstable and prone to degrade in an alkaline environment, free CUR was rapidly cleared by enzymes in the body. The NP encapsulation can protect CUR from being cleared, thus extending the retention time of the drug in the blood. Hence, the bioavailability of CUR was substantially enhanced. Of note, both HA and CS have good biocompatibility and biodegradability and will not increase extra systemic cytotoxicity.

### 3.6. In Vivo Antitumor Studies

**Figure 7:**
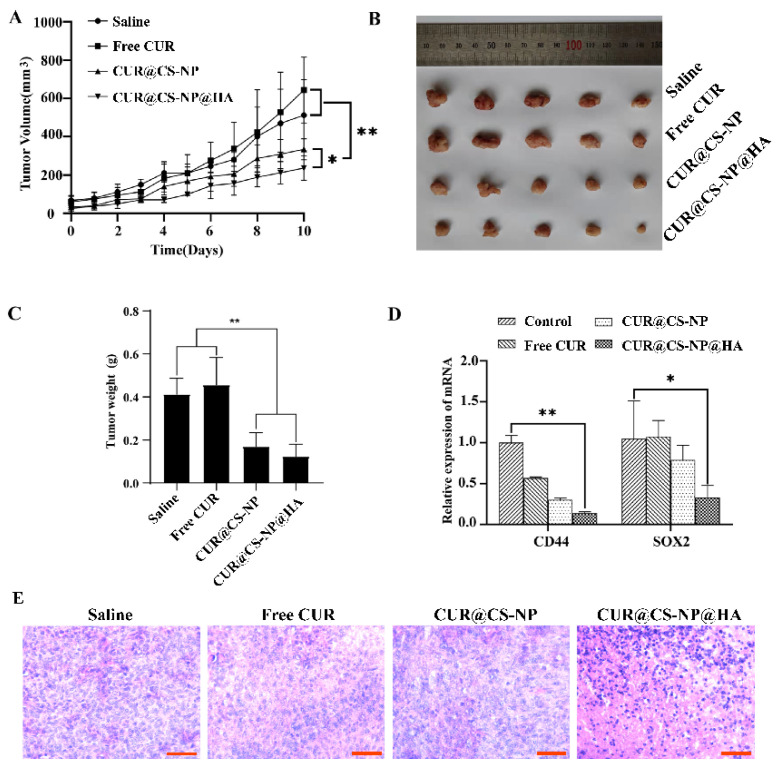
In vivo antitumor potency study. (**A**) Tumor volume growth curve during treatment. (**B**) Representative tumor photos from the different treatment groups. (**C**) Final tumor weights after 10 days of treatment. (**D**) Expression of stemness indicators in 4T1 primary tumors (* *p* < 0.05; ** *p* < 0.01). (**E**) Representative HE examinations of tumor tissues (scale bar = 51 µm).

In this section, we sought to investigate the in vivo antitumor activity of the NPs. A mouse model of breast cancer was established by orthotopically inoculating 4T1 tumor cells in Balb/c mice. When the tumor volumes reached 100 mm^3^, saline, free CUR, CUR@CS-NP, and CUR@CS-NP@HA were administered intravenously. Compared with free CUR, the tumor growths of CUR@CS-NP and CUR@CS-NP@HA-treated mice were substantially slower (Figure 7A–C). Of note, all treatments were well tolerated, without obvious body weight loss (Figure S10) and without obvious damages to heart, liver, spleen, lung, and kidney, evidenced by HE staining (Figure S11). As such, CUR@CS-NP@HA successfully augmented CUR’s therapeutic efficacy of inhibiting tumor growth in a safe therapeutic window.

Further studies were performed to disclose the underlying mechanisms of CUR@CS-NP@HA against tumor growth. Stemness-related proteins, SOX2 and CD44, are usually involved in the signal transduction pathways that control cell proliferation, migration, invasion, stemness, tumorigenesis, and anti-apoptosis. qPCR assays were performed to explore the relative mRNA expressions of CD44 and SOX2 in mouse tumor tissues. CUR@CS-NP@HA could significantly reduce the mRNA expression of CD44 and SOX2 in tumor tissue compared with the free CUR group (Figure 7D). Moreover, more necrosis areas were observed in the CUR@CS-NP@HA-treated tumors (Figure 7E), which is also indicative of the augmented therapeutic efficacy of CUR@CS-NP@HA.

Overall, in a mouse model, CUR@CS-NP@HA exhibited great potential to treat breast cancer, such as inhibiting primary tumor growth, and downregulating stemness-related mRNAs.

### 3.7. Blood Routine Analysis

Over 10 days administration of CUR@CS-NP@HA, the venous blood of mice was collected for routine blood tests (Table 6). The lower levels of white blood cells indicated that the NPs could partially avoid the inflammatory response. Other results were all within the normal range, indicating that the tail vein injection of NPs did not cause significantly hostile changes in blood related indexes, and the NPs had good biocompatibility with the mouse circulation system.

## 4. Conclusions

A facile HA-coating strategy through a self-assembly manner was successfully developed to deliver curcumin for breast cancer treatment. HA could readily attach to the CUR@CS_HMW_-NP core through electrostatic interactions. This method avoids the conventional chemical manipulation of the HA structure, which is particularly sophisticated and hinders excessive applications. This NP system displayed a moderate size, good stability, pH-sensitive properties, and enhanced therapeutic efficacy against breast cancer compared to the plain counterpart, all of which identified the successful HA anchorage at the NP surface. Therefore, the present study provides a novel strategy for HA anchoring as the CD44-targeting ligand and will be a good reference for small-molecule treatment in the clinic.

## Figures and Tables

**Table 1:** Independent variables and levels of experimental design.

Independent Variables	Levels		
	−1	0	1
X_1_: CUR/CS-*g*-PN ratio (*w*/*w*)	1:5	1:7	1:9
X_2_: pH	4	5	6
X_3_: CUR dosage (mg)	3	5	7

**Table 2:** Primer sequences.

Gene	Primer Sequence	Base Number
M-GAPDH-F	CAGGAGAGTGTTTCCTCGTCC	21
M-GAPDH-R	TTCCCATTCTCGGCCTTGAC	20
M-SOX2-F	CTCGCAGACCTACATGAAC	19
M-SOX2-R	CTCGGACTTGACCACAGA	18
M-CD44-F	CACCTTGGCCACCACTCCTAAT	22
M-CD44-R	CCCTTCTGTCACATGGGAGTC	21

**Table 3:** Predicted value and observed value of optimal preparation process of CUR@CS-NP.

Parameter	Predicted Value	Measured Value
PS (nm)	229.09	213.90 ± 6.0
EE%	67.86	70.20 ± 3.90
DL%	11.65	11.40 ± 1.50

**Table 4:** Size, drug loading, and encapsulation rates of different NPs.

NPs	PS (nm)	EE%	DL%
CUR@CS_LMW_-NP	245 ± 6.5	62.81 ± 2.35	10.47 ± 0.39
CUR@CS_MMW_-NP	266 ± 12.3	51.46 ± 3.40	8.58 ± 0.57
CUR@CS_HMW_-NP	212 ± 5.0	69.10 ± 2.00	11.35 ± 0.33

**Table 5:** Pharmacokinetic parameters of CUR@CS-NPs (1.5 mg/kg) by tail vein injection in rats.

Pharmacokinetics	CUR Solution	CUR@CS-NP	CUR@CS-NP@HA
AUC (0–∞) (h × ng/mL)	502.30 ± 123.80	3133.13 ± 276.90	1979.29 ± 373.01
MRT (0–∞) (h)	0.41 ± 0.07	31.28 ± 8.76	6.48 ± 1.90
t1/2 (h)	0.58 ± 0.29	12.52 ± 0.89	9.88 ± 6.33
CL (mL/h/kg)	0.004 ± 0.001	0.003 ± 0.01	0.001 ± 0.00
Tmax (h)	0.04 ± 0.02	0.05 ± 0.02	0.04 ± 0.02
Cmax (ng/mL)	694.11 ± 128.96	522.06 ± 256.11	609.76 ± 87.54

**Table 6:** After treatment, routine blood analysis of mice in each group.

Index	Saline	Free CUR	CUR@CS-NP	CUR@CS-NP@HA
WBC (10^9^/L)	56.85 ± 21.42	67.72 ± 19.78	71.90 ± 26.59	16.77 ± 12.63
RBC (10^12^/L)	9.44 ± 0.32	9.91 ± 0.48	7.98 ± 0.95	8.14 ± 0.65
HGB (g/L)	144.60 ± 3.71	153.60 ± 6.95	112.20 ± 13.85	114.40 ± 8.44
HCT (%)	43.54 ± 1.15	45.68 ± 2.41	34.50 ± 4.31	35.06 ± 2.43
MCV (fL)	46.14 ± 0.34	46.14 ± 0.74	43.20 ± 0.78	43.30 ± 1.70
MCH (pg)	15.34 ± 0.40	15.52 ± 0.15	14.04 ± 0.29	14.12 ± 0.61
MCHC (g/L)	332.20 ± 7.72	336.40 ± 6.07	325.20 ± 4.76	326.20 ± 4.09
RDW (%)	16.40 ± 0.68	16.74 ± 0.87	18.60 ± 1.03	18.40 ± 1.03

## Data Availability

Not applicable.

## Supplementary Materials

The following supporting information can be downloaded at: https://www.mdpi.com/article/10.3390/pharmaceutics14112461/s1.
