# Supplementary material for: Self−Assembling Anchorage of Hyaluronic Acid on the Nanoparticle Surface Confers Superiority of Triple Negative Breast Cancer Treatment
Source: Pharmaceutics. 2022 Nov 15;14(11):2461. doi: 10.3390/pharmaceutics14112461 (PMC9695327; doi:10.3390/pharmaceutics14112461)
Supplement: Supplementary file 1 [file pharmaceutics-14-02461-s001.zip › pharmaceutics-1977185-supplementary.pdf]

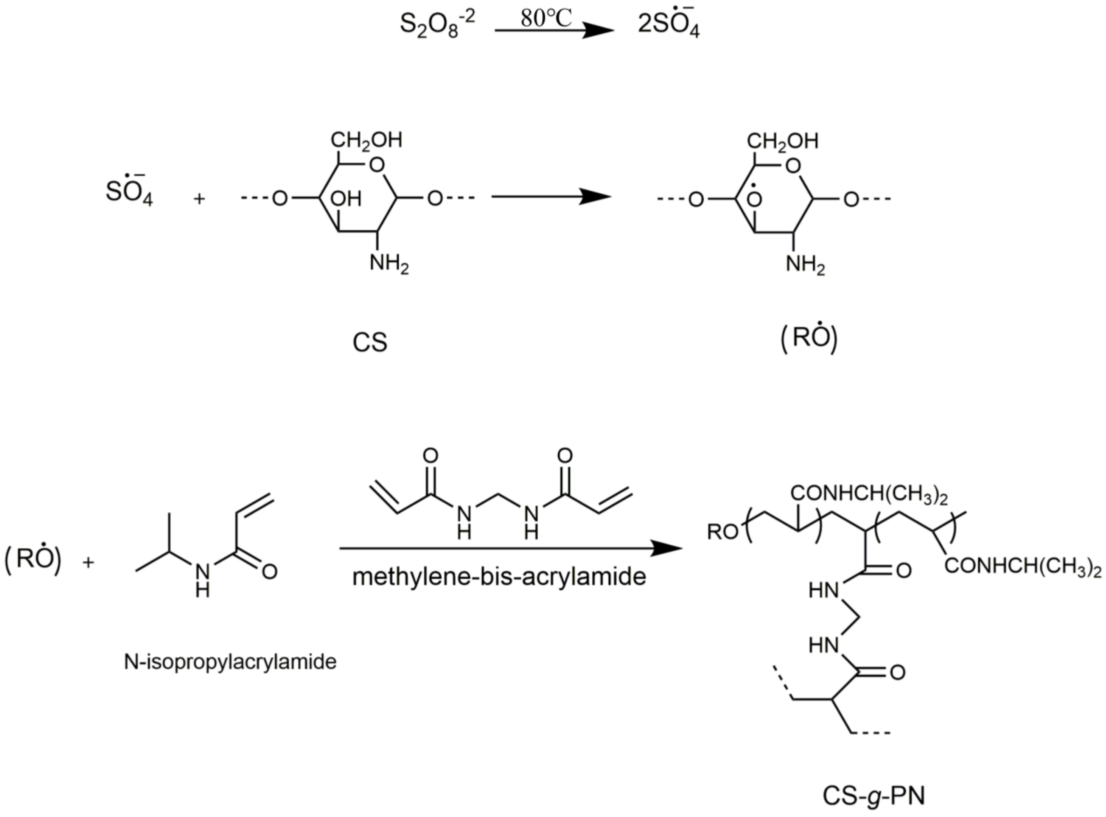

Figure S1. Schematic synthesis of CS−*g*−PN.

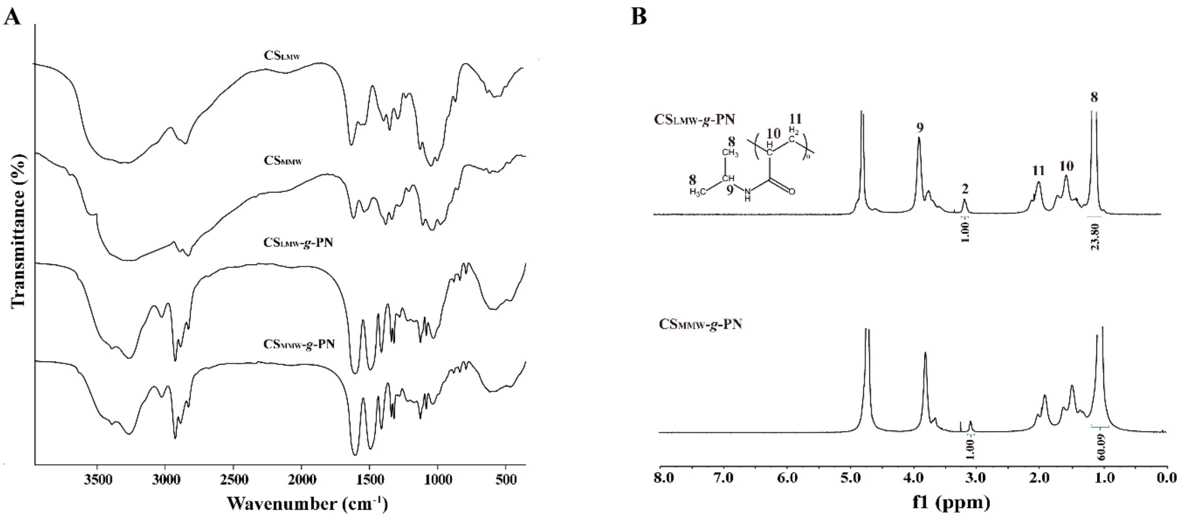

Figure S2. Characterization of CS–*g*–PN. (A) FT–IR spectrum of CS_{LMW}, CS_{MMW}, CS_{LMW}–*g*–PN and CS_{MMW}–*g*–PN. (B) ^{1}H–NMR spectrum of CS_{LMW}–*g*–PN and CS_{MMW}–*g*–PN.

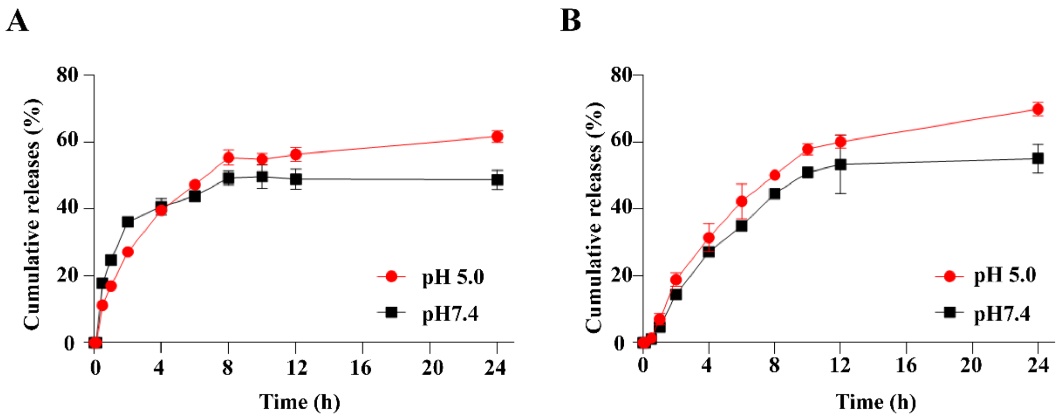

Figure S3. In vitro release profile of CUR@CS–NP. (A) $CUR@CS_{LMW}$–NP (B) $CUR@CS_{MMW}$–NP.

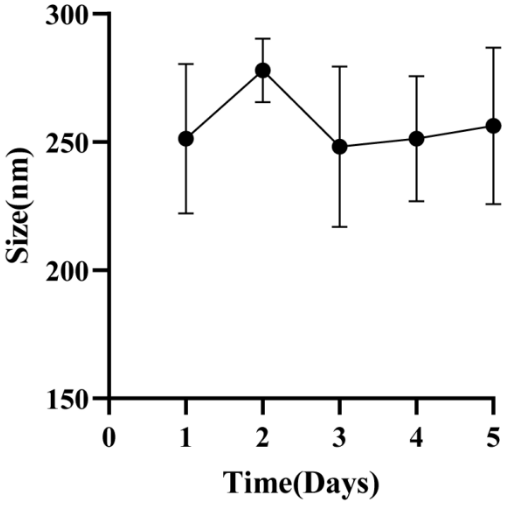

Figure S4. Colloidal stability of CUR@CS−NP@HA within 5days.

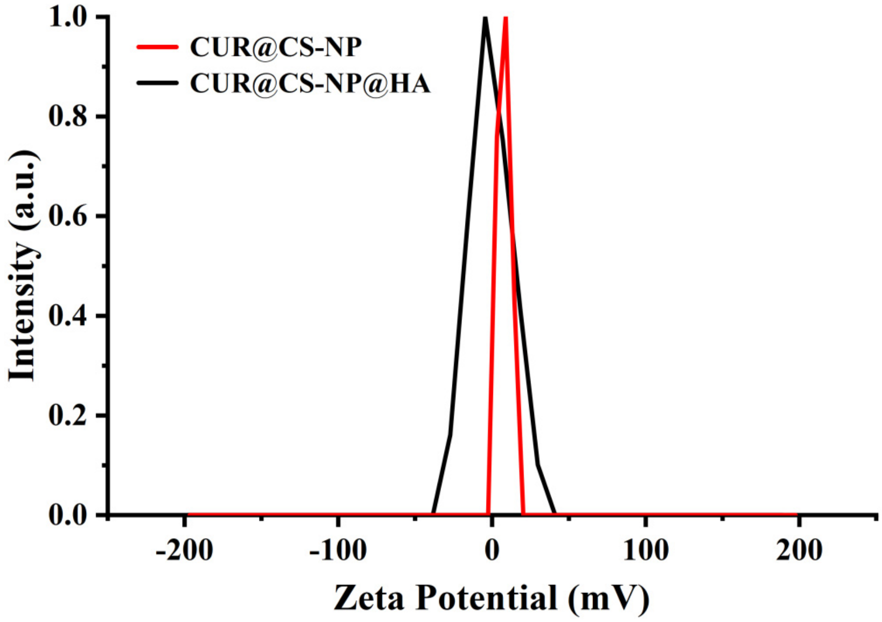

Figure S5. Zeta Potential distributions of CUR@CS−NP and CUR@CS−NP@HA.

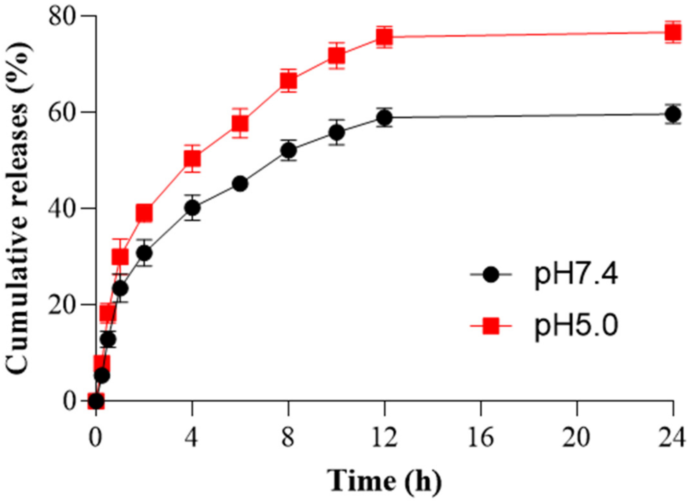

Figure S6. In vitro release profile of CUR@CS−NP@HA.

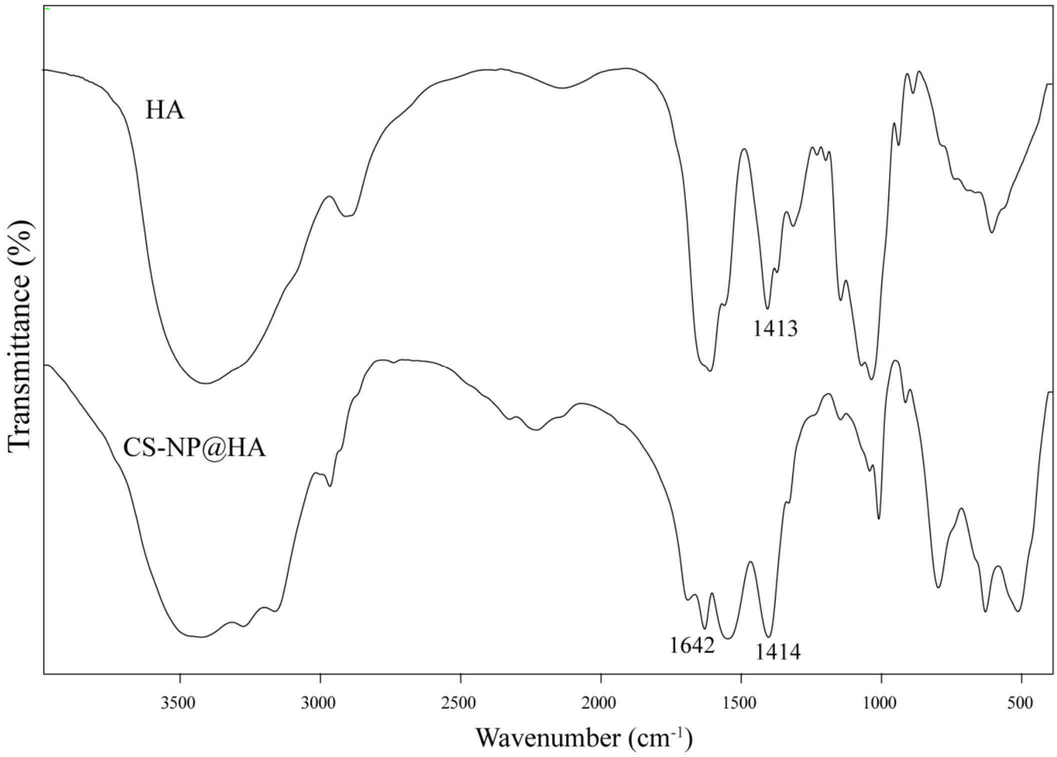

Figure S7. FT−IR spectra of HA and CS−NP@HA. Different from FT−IR spectrum of CS−*g*−PN, the amide bond absorption peak of CS at CS−NP@HA shifts to 1642 cm^{-1}, and the C=O (−COOH) symmetric stretching vibration peak of HA shifts from 1413 cm^{-1} to 1414 cm^{-1}, all of which indicates HA interacting with CS−*g*−PN.

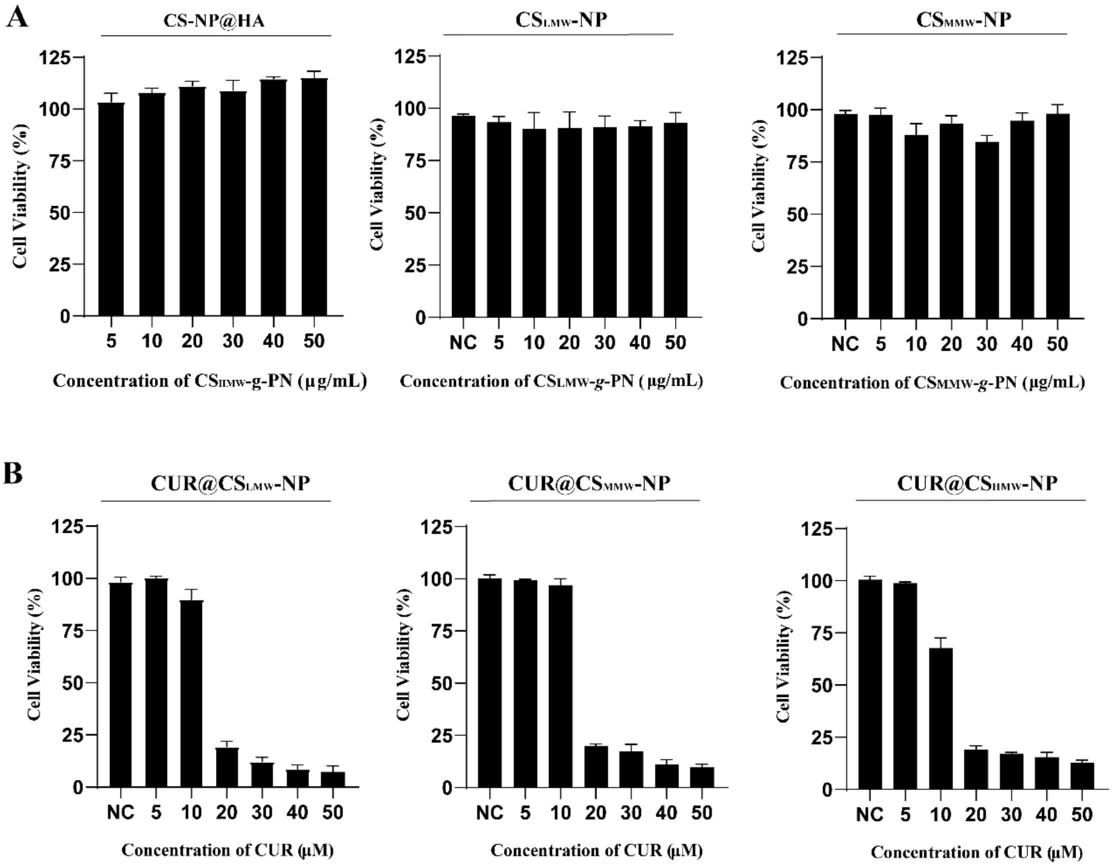

Figure S8. Cell cytotoxicity assay. (A) Cytotoxicity of blank NPs. (B)Cytotoxicity of CUR@CS_{LMW}–NP, CUR@CS_{MMW}–NP and CUR@CS_{HMW}–NP.

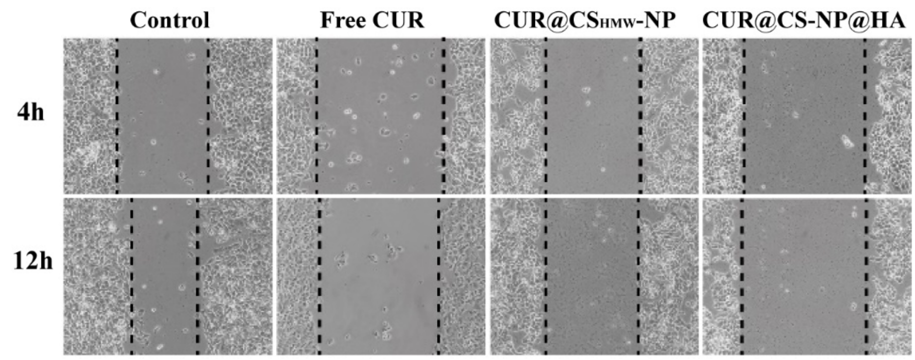

Figure S9. The migration of 4T1 cells treated with NPs in 4h and 12h.

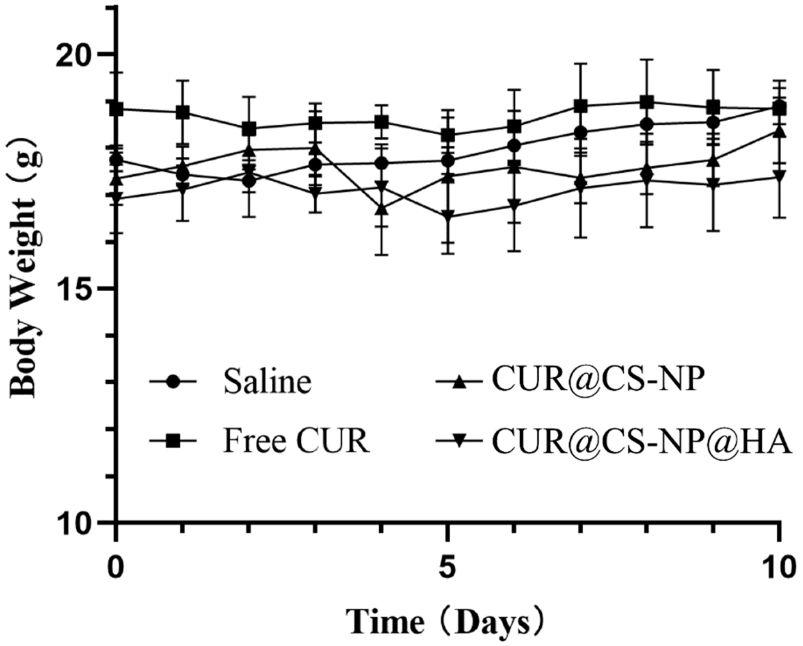

Figure S10. Body weight change in Balb/c mice over 10 days.

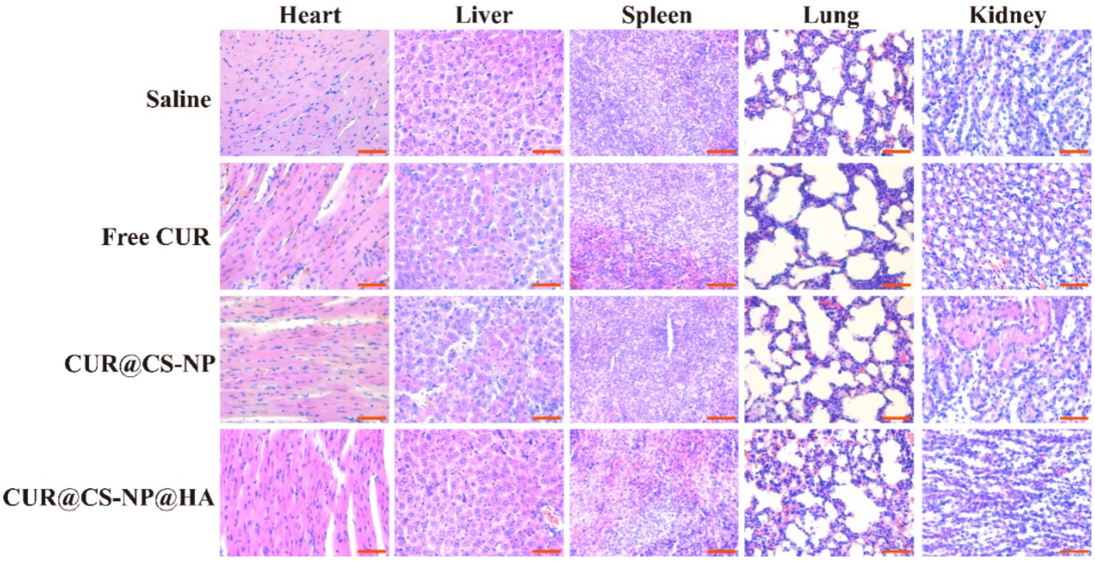

Figure S11. After 10 days of treatment, HE staining of heart, liver, spleen, lung and kidney tissues were taken from tumor–bearing Balb/c mice (scale bar=51μm).
